# Using different zebrafish models to explore liver regeneration

**DOI:** 10.3389/fcell.2024.1485773

**Published:** 2024-10-31

**Authors:** Dashuang Mo, Mengzhu Lv, Xiaoyu Mao

**Affiliations:** ^1^ Department of Immunology, College of Basic Medical Sciences, Guizhou Medical University, Guiyang, Guizhou, China; ^2^ College of Language Intelligence, Sichuan International Studies University, Chongqing, China

**Keywords:** zebrafish, liver injury, biliary epithelial cell, hepatocyte, regeneration

## Abstract

The liver possesses an impressive capability to regenerate following various injuries. Given its profound implications for the treatment of liver diseases, which afflict millions globally, liver regeneration stands as a pivotal area of digestive organ research. Zebrafish (*Danio rerio*) has emerged as an ideal model organism in regenerative medicine, attributed to their remarkable ability to regenerate tissues and organs, including the liver. Many fantastic studies have been performed to explore the process of liver regeneration using zebrafish, especially the extreme hepatocyte injury model. Biliary-mediated liver regeneration was first discovered in the zebrafish model and then validated in mammalian models and human patients. Considering the notable expansion of biliary epithelial cells in many end-stage liver diseases, the promotion of biliary-mediated liver regeneration might be another way to treat these refractory liver diseases. To date, a comprehensive review discussing the current advancements in zebrafish liver regeneration models is lacking. Therefore, this review aims to investigate the utility of different zebrafish models in exploring liver regeneration, highlighting the genetic and cellular insights gained and discussing the potential translational impact on human health.

## 1 Introduction

Liver diseases encompass a range of conditions affecting the liver, including hepatitis, cirrhosis, liver cancer, and fatty liver disease. These illnesses can significantly impair liver function, often leading to severe health complications and, in advanced stages, liver failure (Wang et al., 2014; Xiao et al., 2019). Given the central roles of the liver in detoxifying the blood, aiding digestion, and regulating metabolism (Miyajima et al., 2014; Michalopoulos and Bhushan, 2021), maintaining its health is critical.

Liver regeneration is a remarkable natural process where the liver regenerates lost or damaged tissue, a vital capability for survival after injury or surgical removal of the liver. Unlike most organs, the liver can regenerate to its full size and function from as little as 30% of its original mass (Gilgenkrantz and de l’Hortet, 2018; Yagi et al., 2020). This regeneration involves complex interactions among various types of cells and signaling pathways (Kiseleva et al., 2021; Michalopoulos and Bhushan, 2021). Understanding these mechanisms is crucial for developing treatments for liver diseases, as enhancing the liver’s natural regenerative abilities could potentially reverse or mitigate the effects of liver conditions. Researchers often study model organisms, such as mice and zebrafish, to gain insights into the cellular and molecular foundations of liver regeneration, aiming to apply these findings to improve human health (Forbes and Newsome, 2016; Van Haele et al., 2019).

Zebrafish are increasingly used as a model organism in regenerative medicine research, particularly for studying organ regeneration (Wang et al., 2017; Marques et al., 2019). This small tropical fish possesses remarkable regenerative abilities, capable of repairing and regrowing several organs and tissues, including the heart, spinal cord, retina, and liver, even as adults (Marques et al., 2019). The high regenerative capacity of zebrafish offers valuable insights into the biological mechanisms that could be harnessed to improve regenerative therapies in humans. Moreover, genes are highly conserved between humans and zebrafish, making it a valuable system for studying the basic mechanisms of liver disease (Goessling and Sadler, 2015; Wrighton et al., 2019; Wang et al., 2021).

**FIGURE 1:**
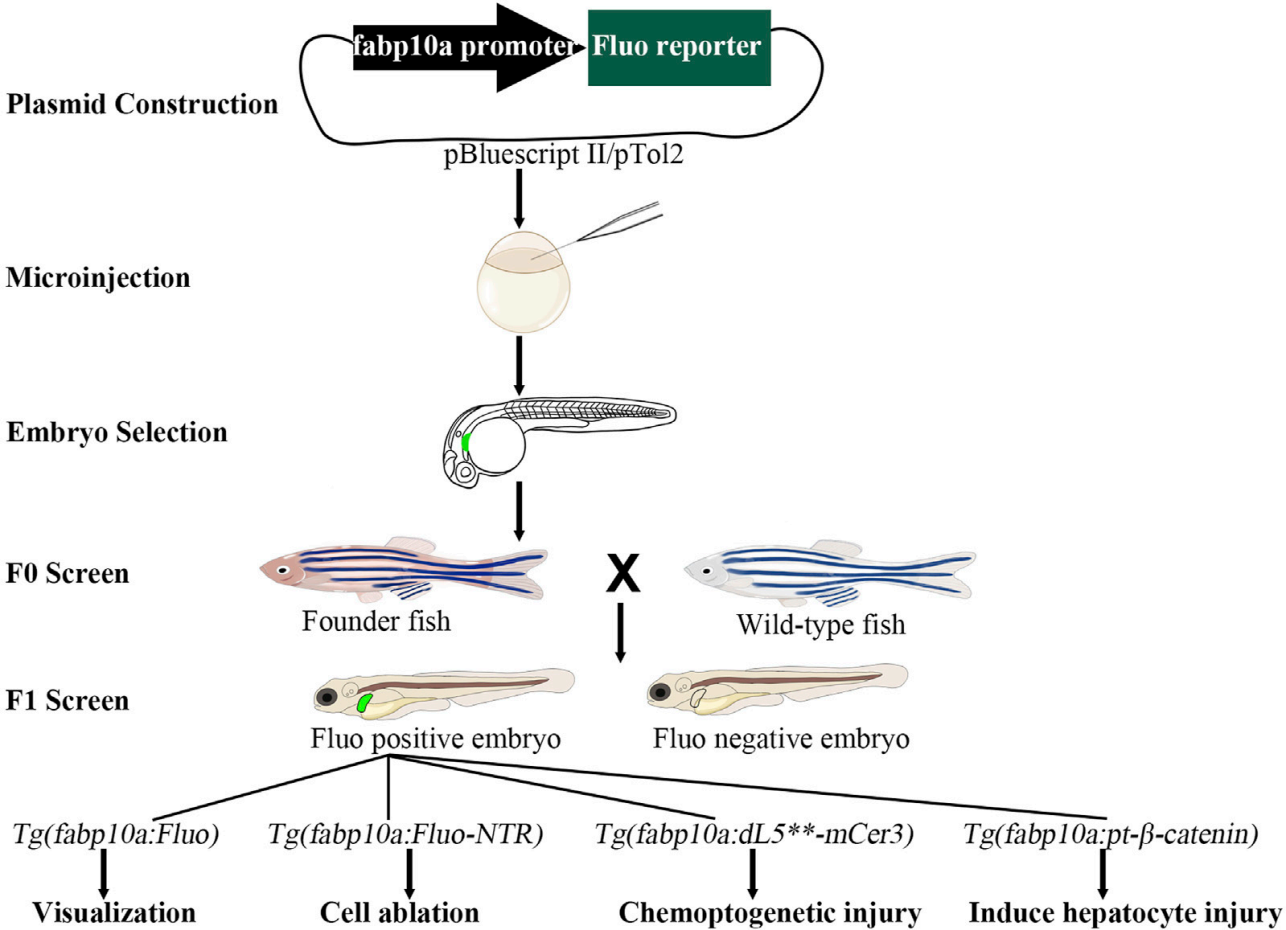
The construction of transgenic models in zebrafish. The fluorescent reporter genes are initially linked to the *fabp10a* promoter in the pBluescript II or pTol2 vector. The resulting plasmid is then microinjected into one-cell stage embryos. At 3–5 days post-fertilization, embryos expressing fluorescent proteins are selected for further development. At the adult stage, founder (F0) fish are crossed with wild-type fish to produce F1 embryos. Further crossing of F1 fish with wild-type fish results in the establishment of a stable transgenic line. These transgenic lines can be utilized for applications such as visualization, cell ablation, chemoptogenetic injury, and induced hepatocyte injury studies. Fluo, fluorescent.

Except for the Kupffer cell, the zebrafish liver contains all other cell types of the mammalian liver (Goessling and Sadler, 2015; Cai et al., 2021). Zebrafish are similar to mammals in hepatic cellular composition, function, signaling, and response to injury, as well as the cellular processes that mediate liver diseases. Importantly, all the liver functions, including bile secretion, glycogen and lipid storage, insulin responsiveness, xenobiotic and ammonia metabolism, and secretion of serum proteins, are fulfilled in zebrafish liver as early as 5 days post-fertilization (dpf) (Goessling and Sadler, 2015). Compared to mammalian models such as mice and monkeys, zebrafish presents several advantages for studying liver regeneration, including embryonic transparency, rapid growth rate, genetic manipulability, and conserved regulatory mechanisms (MacRae and Peterson, 2015; Huang et al., 2016; van der Helm et al., 2018). Zebrafish have become a widely used model for studying liver regeneration, primarily due to the development of several liver-specific transgenic lines (Figure 1). Research on zebrafish liver regeneration focuses on three models: partial hepatectomy, drug-induced liver injury, and genetic ablation. This review will discuss the current findings of these three frequently used and three other uncommon zebrafish models, especially the genetic ablation model that first uncovered the process of biliary-to-hepatocyte transition, and summarize the regulatory factors involved in these regenerative processes (Table 1).

**TABLE 1:** Comparison of liver injury and regeneration using different zebrafish models.

Zebrafish liver injury models	Transgenic lines used	Cell sources for hepatocyte regeneration	Critical regulatory factors
Partial hepatectomy	*Tg(fabp10a:CFP-NTR)*	Uninjured hepatocytes	BMP, FGF, Wnt, Uhrf1, Top2a, Def, Capn3, p53, TGF-β, Chk1, Wee1, GSNOR
Genetic ablation	*Tg(fabp10a:Dendra2-NTR)* *Tg(fabp10a:mCherry-NTR)* *Tg(fabp10a:CFP-NTR)*	Biliary epithelial cells	mTOR, PI3K, VEGF, Dnmt1, BET, Tel2, FXR, Mdka, ERK1, p53, Hhex, Bmp, Hdac1, Wnt, Notch, Myca, Sox9b, Cdk8, Fbxw7, MRN, Stat3, Epcam
Drug-induced liver injury	*Tg(fabp10a:GFP)*	Uninjured hepatocytes	BET, Nrf2, FXR
Chemoptogenetic liver injury	Tg(fabp10a:dL5**-mCer3)	Uninjured hepatocytes and biliary epithelial cells	None
Oncogene overexpression-induced liver injury	*Tg(fabp10a:pt-β-catenin)*	Uninjured hepatocytes and biliary epithelial cells	EGFR, ERK1/2, Sox9, PPARα, Yap
Hepatic cryoinjury	*Tg(fabp10a:NLS-mCherry)*	Uninjured hepatocytes	None

## 2 Liver morphology and structure in zebrafish

Despite the high conservation of cell types within the liver, zebrafish possess a unique hepatic anatomy and cellular architecture compared to mammals. The adult zebrafish liver is composed of three contiguous lobes (two lateral and one ventral), which lack the pedicle that separates distinct lobes in mammalian livers. Instead of a portal architecture, fish livers feature hepatocytes arranged in tubules, with bile ductules running between two rows of hepatocytes. The apical membranes face the interior of the tubule, while sinusoids follow the basal side of hepatocytes (Goessling and Sadler, 2015). From the ScRNA-seq data, several cell types were identified in zebrafish livers, including hepatocytes, biliary epithelial cells (BECs), vascular endothelial cells, blood cells, and hepatic stellate cells (Cai et al., 2021). The only difference between the cell types of zebrafish and mammalian livers is the existence of hepatic immune cells (such as Kuffer cells), which could not detected in normal zebrafish livers (Goessling and Sadler, 2015). Fish usually possess two types of BECs: small preductal BECs, which form intracellular lumens to transport bile from hepatocytes, and larger columnar cholangiocytes, which construct the complete intrahepatic biliary system (Weis, 1972; Hampton et al., 1988; Hampton et al., 1989). The formation of small preductal BECs was revealed by time-lapse imaging of living zebrafish larvae, in which bile duct lumen occurs typically through the fusion of cytoplasmic vesicles between two adjacent BECs (Lorent et al., 2010). Even the fact that zebrafish owns unique liver structure, the high conservation of genes between humans and zebrafish makes zebrafish a valuable model for studying the fundamental mechanisms of liver injury and regeneration.

## 3 Partial hepatectomy (PH) in zebrafish

Since the liver weight varies between individuals, the standard parameter used to study zebrafish liver regrowth is the liver/body weight ratio (L/B ratio), which is constant within other species (Weglarz and Sandgren, 2000). Likewise, despite the fact that a difference in L/B ratio between males and females had been observed in adult zebrafish, the L/B ratio in the same gender is constant and does not depend on age (Kan et al., 2009), making it easy to evaluate the efficiency of liver regeneration.

### 3.1 Model construction

The adult zebrafish liver is structurally different from the mammalian liver. It contains three liver lobes, two dorsal lobes, and a single ventral lobe flattened along the intestine (Unterweger et al., 2023). Unlike the 2/3 PH in mammals, the resection of any two liver lobes led to intense bleeding, disrupted blood circulation, and subsequent mortality (Kan et al., 2009). Thus, the zebrafish PH model is characterized as a 1/3 partial hepatectomy in which the entire ventral lobe is surgically removed (Oderberg and Goessling, 2021).

**FIGURE 2:**
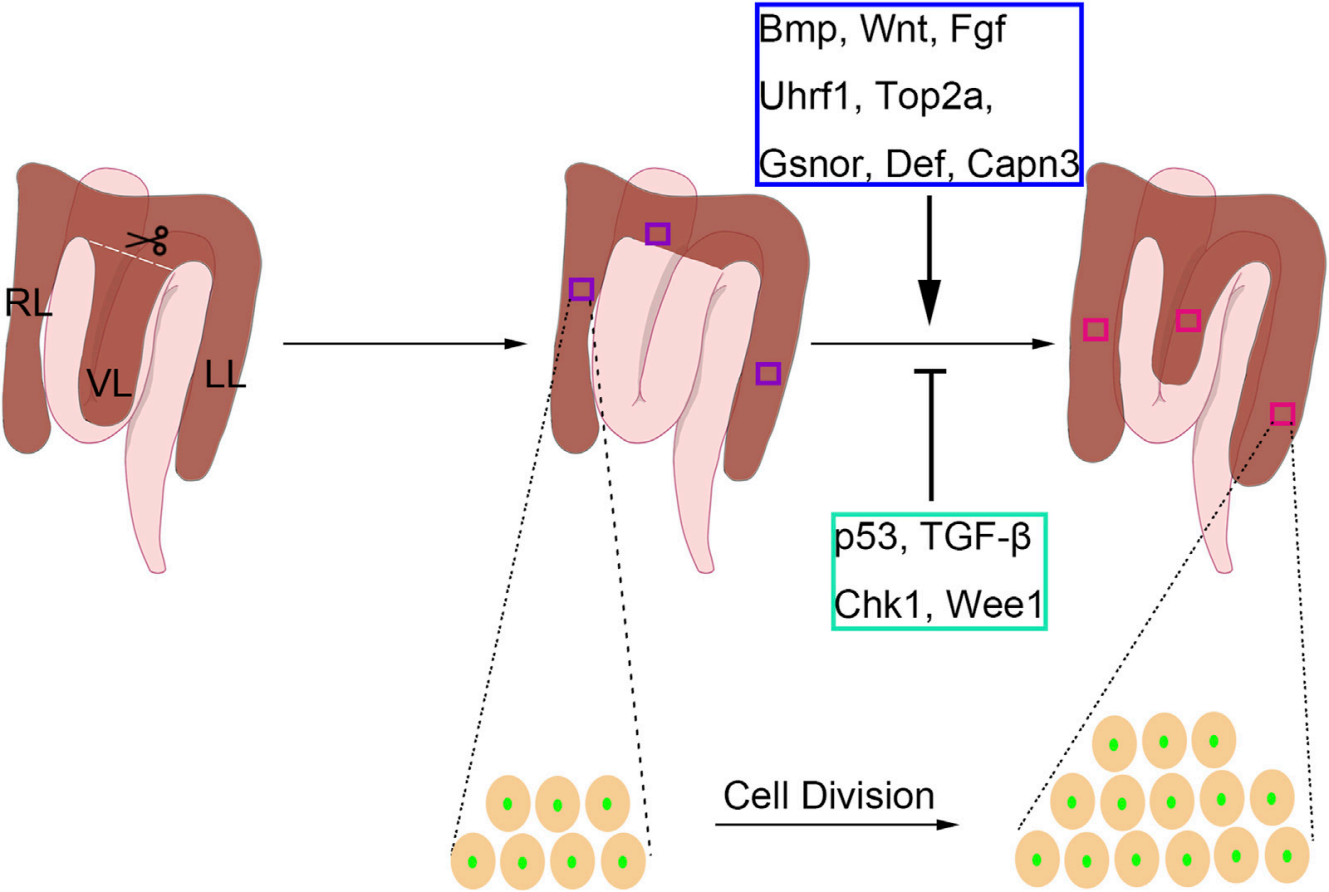
The partial hepatectomy (PH) model in adult zebrafish. In the zebrafish one-third PH model, the entire ventral lobe (VL) is removed, and the left lobe (LL) and right lobe (RL) are retained. The local regeneration of VL and compensatory regeneration of LL and RL are both present upon PH-induced liver injury in adult zebrafish. The liver regenerative types differ from different PH protocols. The liver regeneration is achieved by intrahepatic cell division. During the regenerative process, Bmp, Wnt, Fgf, Uhrf1, Gsnor, Def, and Capn3 play positive roles, while p53, TGF-β, Chk1, and Wee1 have negative roles.

After the PH surgery, the L/B ratio would be immediately reduced to approximately 65% of uninjured fish, indicating that the injury model is a 1/3 hepatectomy (Figure 2). The liver mass would be recovered to the average level 7 days post-surgery, as reflected by the L/B ratio. Over the next 2–3 days, an additional increase of L/B ratio (10% higher than normal fish) would be observed. After this, the L/B ratio decreased to 90% at 14 days post-surgery and then normalized at 28 days post-surgery (Kan et al., 2009). This pattern of liver regeneration in zebrafish is similar to mammalian liver regeneration, marked by an initial increase in liver mass, followed by a slow return to the original liver mass (Michalopoulos and DeFrances, 1997). Despite the L/B ratio presenting gender differences, males and females show similar regenerative dynamics after surgery (Kan et al., 2009).

### 3.2 Regenerative process

Like the mammalian PH model (Michalopoulos and Bhushan, 2021), liver regeneration in the zebrafish PH model is achieved by the contribution of uninjured hepatocyte proliferation. Lineage tracing data reveal that BECs fail to contribute to hepatocyte regeneration after PH in zebrafish (Zhang et al., 2021), indicating the conserved regenerative process between zebrafish and mammals. However, zebrafish liver regeneration after PH could be divided into two ways: local regeneration and compensatory regeneration.

After 1/3 PH surgery in zebrafish liver, the regeneration of the missing ventral lobe could result in compensatory regeneration in the dorsal lobes other than the ventral lobe and ultimately lead to the recovery of liver mass within a week (Kan et al., 2009; Oderberg and Goessling, 2021). The compensatory growth of the liver after hepatectomy of the ventral lobe occurs in zebrafish, and this process, similar to those in rodents and humans, is closely associated with the activation and proliferation of hepatocytes (Kan et al., 2009). Transcriptomic profiling of the dorsal lobes following resection of the ventral lobe revealed significant changes related to compensatory regeneration (Feng et al., 2015). Meanwhile, another research group reported that the ventral lobe could mostly regenerate over 36 days post-surgery (Sadler et al., 2007), suggesting that the zebrafish owns the capacity for epimorphic liver regeneration, which differs in mammals (Goessling and Sadler, 2015). Considering the liver regeneration models vary with the extent of the injury, the technical variation in the PH protocol between research groups may lead to discrepancies in results.

### 3.3 Regulatory factors involved in PH-induced zebrafish liver regeneration

In mammals, several signaling pathways have been shown to regulate PH-induced liver regeneration, including BMP, FGF, and Wnt signaling (Forbes and Newsome, 2016), which are also involved in regulating liver development (Zong and Stanger, 2012). Similar results were carried out using the zebrafish PH model. By using the zebrafish transgenic lines *Tg(hsp:dnBPMR-GFP)* and *Tg(hsp:dnFGFR1-GFP)*, BMP and FGF were proved to be essential for hepatocyte proliferation after PH in both males and females (Kan et al., 2009). The Wnt/β-catenin pathway was shown to be activated after PH (Kan et al., 2009), implying that Wnt signaling also regulates liver regeneration in zebrafish. Indeed, Wnt activation by Wnt8a overexpression would promote PH-induced liver regeneration in zebrafish, while Wnt inhibition by dnTCF expression would reduce the regenerated liver mass (Goessling et al., 2008). Because PH-induced liver regeneration in zebrafish is achieved by hepatocyte proliferation, the modulation of cell cycle regulators could affect the regenerative process. Uhrf1 regulates the outgrowth of the developmental liver by inducing the genes involved in the cell cycle. Besides, *uhrf1* heterozygous mutants exhibited defective regeneration after PH, indicating the essential roles of Uhrf1 in regulating the cell cycle upon acute liver injury (Sadler et al., 2007). Top2a has traditionally served as a marker for proliferation in both normal and cancer tissues, and Uhrf1 is recognized as a known positive regulator of Top2a activity (Wang et al., 1997; Hopfner et al., 2000; Zhu et al., 2002). Another study reported that *top2a* heterozygosity also caused the deficiency of liver regeneration in adult zebrafish (Dovey et al., 2009), suggesting that the promotion of Uhrf1-Top2a axis in cell proliferation is essential for liver regeneration in the PH model. The nucleolus complex Def-Capn3 also participates in PH-induced liver regeneration in zebrafish. Haploinsufficiency of def activates p53-dependent TGF-β signaling and causes scar formation after PH (Zhu et al., 2014), and Def-Capn3 complex the cell cycle reentry of hepatocytes by inhibiting Chk1 and Wee1 during liver regeneration (Chen et al., 2020). In addition to these factors, s-nitrosoglutathione reductase (GSNOR) plays opposing roles in PH-induced zebrafish liver regeneration, which was confirmed by the promoted phenotype upon genetic mutation or pharmacological inhibition of GSNOR (Cox et al., 2014).

The regulatory factors revealed in the zebrafish PH model deepen our understanding of hepatocyte proliferation upon hepatectomy (Figure 2). However, most of these findings have been previously reported in mammals, thus limiting the novelty of these studies. Additionally, due to the circulating breed system, the zebrafish PH model could hardly be used for drug treatment and screening. Hence, zebrafish is not a popular model to study PH-induced liver regeneration.

## 4 Genetic ablation of hepatocytes in zebrafish

Since nitroreductase (NTR) could convert the nontoxic prodrug metronidazole (Mtz) to the cytotoxic form, Mtz treatment specifically ablates cells that express NTR. The Mtz/NTR system is cell-cycle independent and applicable to any target cell population (Bridgewater et al., 1995). The NTR enzyme is initially reduced by NADH or NADPH. Following this reduction, NTR binds to Mtz, reducing it to a powerful DNA interstrand cross-linking agent, leading to cell death (Curado et al., 2007). Until now, Mtz/NTR system has been used to study several organ or tissue regeneration in zebrafish, such as cerebrovascular, retina, heart, liver, pancreas, and fin (Gemberling et al., 2013; Chen et al., 2019; Mi et al., 2024).

### 4.1 Model construction

**FIGURE 3:**
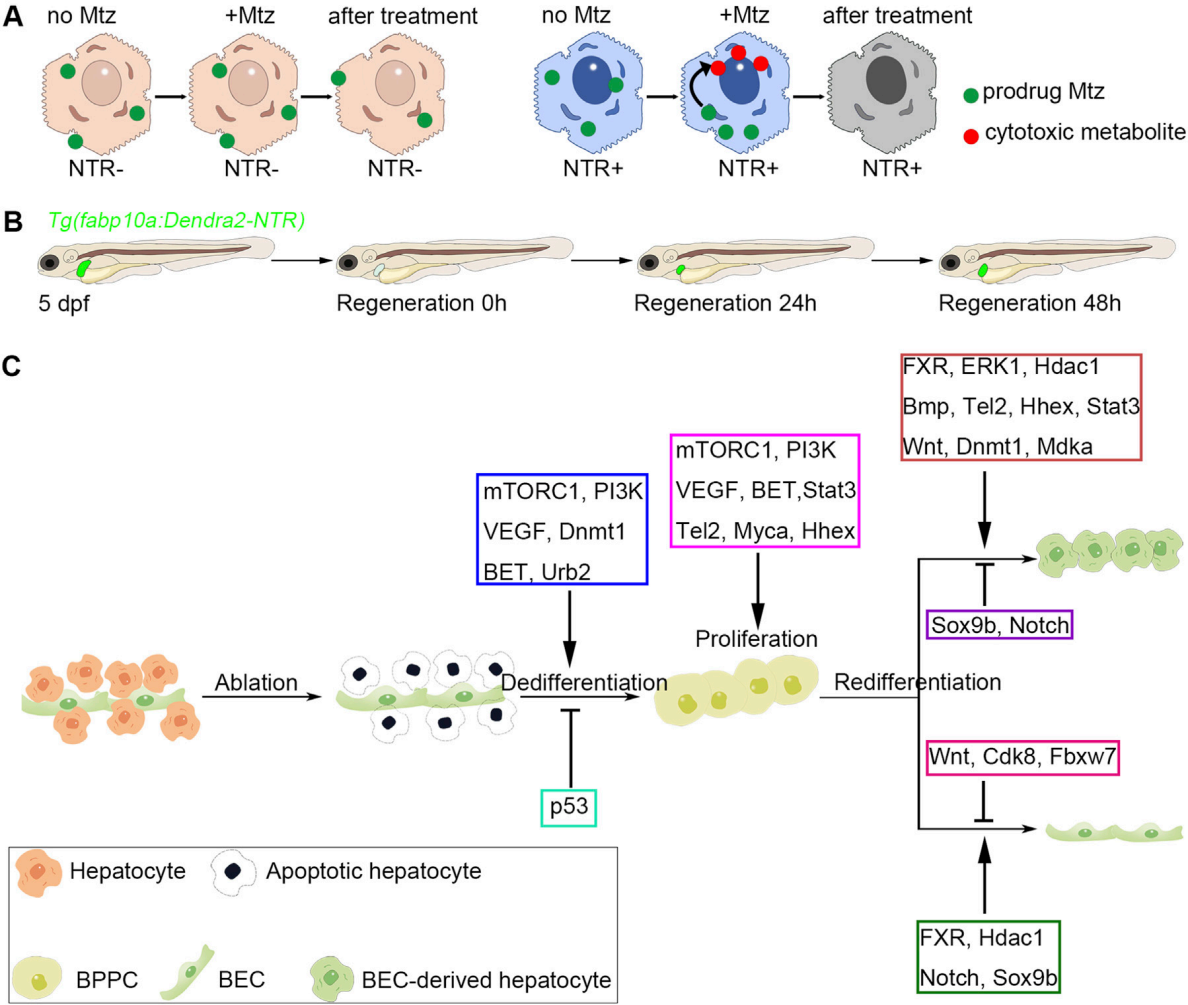
Genetic ablation of hepatocytes and biliary-mediated liver regeneration in zebrafish. **(A)** The work model of Mtz/NTR system in zebrafish liver. Nitroreductase (NTR) converts the nontoxic prodrug metronidazole (Mtz) to the cytotoxic metabolite, which could lead to the cell death of hepatocytes. **(B)** The schematic diagram of zebrafish biliary-mediated liver regeneration model. The fluorescent images of regenerating livers could be obtained by a confocal microscope. **(C)** Regulatory factors involved in different regenerative stages. In the zebrafish extreme hepatocyte injury model, nitroreductase (NTR)-expressed hepatocytes are ablated upon metronidazole treatment. Then, the biliary epithelial cells (BECs) would dedifferentiate into bipotential progenitor cells (BPPCs). mTORC1, PI3K, VEGF, Dnmt1, BET, and Urb2 are essential for BEC-to-BPPC dedifferentiation, while p53 activation inhibits the dedifferentiation process. BPPCs proliferate rapidly after BEC dedifferentiation, and mTORC1, PI3K, VEGF, BET, Stat3, Tel2, Myca, and Hhex positively regulate BPPC proliferation. Lastly, BPPCs re-differentiated into hepatocytes and BECs. During the BPPC-to-hepatocyte redifferentiation process, FXR, ERK1, Hdac1, Bmp, Tel2, Hhex, Stat3, Wnt, Dnmt1, and Mdka exert positive roles, while Sox9b and Notch show negative roles. During the BPPC-to-BEC redifferentiation process, FXR, Hdac1, Notch, and Sox9b play positive roles, while Wnt, Cdk8, and Fbxw7 exhibit negative roles.

Based on the Mtz/NTR system, the transgenic lines *Tg(fabp10a:Dendra2-NTR), Tg(fabp10a:mCherry-NTR)*, and *Tg(fabp10a:CFP-NTR)* were generated by two research groups (Choi et al., 2014; He et al., 2014; Choi et al., 2015). The hepatocytes of these three lines express fluorescent proteins that could be observed by fluorescent microscopes, and the hepatocytes can also be ablated by Mtz treatment (Figure 3). After 10 mM Mtz treatment for 24 h, nearly all hepatocytes would suffer from apoptosis, leading to extreme liver injury in zebrafish. In larval zebrafish, the injured livers could be functionally recovered at 48 h post Mtz treatment, making the larval zebrafish an ideal model to study liver regeneration (He et al., 2014). The Mtz/NTR system also works in adult zebrafish, and only 10 h of Mtz treatment could cause extreme hepatocyte ablation and functional regeneration can be achieved 5 days post-injury (He et al., 2014).

In addition to the above three commonly used transgenic lines that could induce extreme hepatocyte injury, another research group reported a new Mtz/NTR-based double transgenic model that could trigger moderate liver injury. This model contains a driver line *Tg(fabp10a:GAL4-VP16,myl7:Cerulean)* and a effector line: *Tg(UAS:NTR-mcherry)* (Jagtap et al., 2020). By crossing these two single lines with each other, hepatocyte-specific expressed GAL4 would activate the expression of UAS downstream genes (Halpern et al., 2008), leading to the hepatocyte-specific expression of NTR. In this model, even 2.5 mM Mtz treatment for 3 h would cause lethality of adult zebrafish. Thus, 1.5 mM Mtz treatment for 3 h was performed to induce hepatocyte injury. A large number of pyknotic nuclei and enucleated cells were detected at 3 h post-injury, suggesting that the hepatocytes were indeed suffering the injury. Unlike the extreme hepatocyte injury model, this model should be categorized as moderate to intermediate. The expression of hepatocyte marker *fabp10a* could always be detected at high levels during the regeneration process (Jagtap et al., 2020), indicating the moderate damage induced by Mtz/NTR system in this model.

### 4.2 Regenerative process

Unlike hepatocyte proliferation-mediated liver regeneration, hepatocyte regeneration upon extreme liver injury is contributed by BEC transdifferentiation, which is validated by the Cre/Loxp-based lineage tracing system (Choi et al., 2014; He et al., 2014). The BEC-mediated liver regeneration consists of three steps: BEC dedifferentiation to bipotential progenitor cells (BPPCs), BPPC proliferation, and BPPC redifferentiation to hepatocytes and BECs. Firstly, extreme loss of hepatocytes leads to alterations of BEC morphologies and induction of the hepatoblast markers such as *hhex* and *foxa3*, indicating the dedifferentiation of BECs to BPPCs. Then, the BPPCs rapidly proliferate, which could be detected by EdU and PCNA staining. Lastly, BPPCs re-differentiate into nascent hepatocytes, which express the mature hepatocyte markers such as *gc*, *bhmt*, and *tfa* (He et al., 2019; Cai et al., 2021; Cai et al., 2023). In adult zebrafish, hepatocyte regeneration is also achieved by BEC transdifferentiation (Choi et al., 2014; He et al., 2014; Oderberg and Goessling, 2023), indicating the conserved regenerative process between larval and adult zebrafish.

In the moderate hepatocyte injury model *Tg(fabp10a:GAL4-VP16,myl7:Cerulean; UAS:NTR-mcherry)*, hepatocytes but not BECs may play the major roles in liver regeneration. Because lineage tracing data were lacking in the model, all the conclusions made by the authors were based on the RNA-seq data (Jagtap et al., 2020). Even though some BEC markers showed induction at 12 h after injury, the changes were not significant, indicating that BECs would not rapidly proliferate upon hepatocyte injury. On the other hand, the continuous high levels of *fabp10a* also suggested that not all hepatocytes were injured, and the proliferation of uninjured hepatocytes could contribute to normal liver regeneration (Jagtap et al., 2020). However, the contribution of BEC transdifferentiation to liver regeneration could not be absolutely excluded, and the application of lineage tracing experiments is the only way to determine the exact sources of hepatocyte regeneration in this model.

### 4.3 Regulatory factors involved in zebrafish BEC-mediated liver regeneration

#### 4.3.1 Factors that regulate BEC dedifferentiation

##### 4.3.1.1 mTORC1

The BEC dedifferentiation initiates the BEC-mediated liver regeneration (He et al., 2019; Cai et al., 2021). The mammalian target of rapamycin complex 1 (mTORC1) signaling is the first pathway that was reported to govern BEC dedifferentiation (He et al., 2019). In the mouse/rat PH-induced liver injury model, PI3K/AKT activates mTOR to regulate the cell cycle and cell proliferation (Chen et al., 2009), and mTOR-dependent phosphorylation and activation of ribosomal S6 protein kinase 1 (S6K) play dominant roles in regulating the cell cycle during liver regeneration (Espeillac et al., 2011). Upon Mtz-induced hepatocyte injury in zebrafish, mTORC1 signaling would be activated in BECs. Chemical inhibition or genetic inactivation of mTORC1 signaling would disrupt the morphological changes of BECs and reduce the expression of BPPC markers. Mechanistically, mTORC1 regulates BEC-mediated liver regeneration through transcriptionally controlling the ribosome biogenesis protein Urb2 (He et al., 2019). The essential roles of mTORC1-Urb2 axis may imply that protein synthesis is the primary force to drive the cell fate conversion of BECs to liver progenitor cells. Indeed, another study also proved the requirement of mTORC1 signaling in zebrafish liver regeneration, and promoting mTORC1 activation may facilitate the BEC-to-hepatocyte transition (Chaturantabut et al., 2019).

##### 4.3.1.2 Dnmt1

DNA methylation, an essential epigenetic mechanism, plays crucial roles in regulating gene expression and organ regeneration (Albogami, 2019). After extreme hepatocyte injury in zebrafish, *dnmt1*, but not *dnmt3a* or *dnmt3b*, was strongly induced in the liver region, and DNA methylation was continuously maintained in the BECs during the regenerative process. Both pharmacological and genetic inhibitions of DNA methylation would disrupt the dedifferentiation process, thus inhibiting the BEC transdifferentiation to hepatocytes (He et al., 2022). Inhibition of DNA methylation would disrupt the maintenance of DNA methylation at the *p53* locus, thus upregulating the transcription of *p53*. The mutation of *p53* could correct the regenerative defects upon DNA methylation inhibition, while *p53* overexpression in BECs would trigger defective liver regeneration. Besides, the activation of mTORC1 signaling was reduced upon the inhibition of DNA methylation (He et al., 2022), thus linking the DNA methylation to mTOR activation in the process of BEC dedifferentiation.

##### 4.3.1.3 VEGF

The well-known role of VEGF signaling is its regulation on angiogenesis, even in normal or pathological conditions (Hu et al., 2014; Simons et al., 2016). In the zebrafish extreme liver injury model, VEGF ligands were mainly secreted by active hepatic stellate cells upon injury, and then the downstream signaling was activated through the receptor VEGFR2 in BECs. VEGF signaling controls the activation of PI3K-mTORC1 axis, which is essential for BEC dedifferentiation (Cai et al., 2023). Another lab also reported the involvement of VEGF signaling in regulating BEC-mediated liver regeneration (Rizvi et al., 2023). Moreover, VEGFA supplement in mice strongly promoted the dedifferentiation of BECs to BPPCs and therefore increased the number of BEC-derived hepatocytes upon liver injury (Rizvi et al., 2023). These two studies prove that VEGF signaling as an important regulator of BEC dedifferentiation and provides VEGF as a potential modulator to treat end-stage liver diseases.

##### 4.3.1.4 BET

Through chemical screening, the inhibitors of bromodomain and extraterminal (BET) proteins were found to suppress BEC dedifferentiation (Ko et al., 2016). The BET protein family members *brd2a*, *brd3a*, *brd3b* and *brd4* would be upregulated in the regenerating livers. BET inhibition could reduce the expression of BPPC markers and the subsequent hepatocyte regeneration. However, once the BET inhibitors were removed, BEC dedifferentiation would be resumed, indicating that temporal BET inhibition does not permanently impair BEC-mediated liver regeneration. Besides, BET proteins are also involved in hepatocyte proliferation in the mouse PH model (Russell et al., 2017), indicating that the BEC transdifferentiation-mediated and hepatocyte proliferation-mediated liver regeneration share some critical regulatory factors.

#### 4.3.2 Factors that regulate BPPC proliferation

After the dedifferentiation of BECs into BPPCs, the proliferation of BPPCs occurs. Because the progenitor cells have higher proliferating capacity (Liu et al., 2019; Long and Huttner, 2022), those genes involved in BEC dedifferentiation often control BPPC proliferation simultaneously. Indeed, previous studies in zebrafish have shown that the deficiency of mTORC1, VEGF, or BET disrupted BPPC formation and proliferation and thereby led to compromised liver regeneration (He et al., 2019; He et al., 2022; Cai et al., 2023). Myca is reported to regulate BPPC proliferation downstream of BET proteins, and *myca* overexpression could partially rescue the defects caused by BET inhibition (Ko et al., 2016). Unlike those three factors, telomere maintenance 2 (Tel2) does not control the BEC dedifferentiation but regulates BPPC proliferation via hematopoietically expressed homeobox (Hhex) in BPPCS. However, the prominent roles of *tel2* are involved in the BPPC redifferentiation (Zhang et al., 2022), which will be discussed in the following section.

#### 4.3.3 Factors that regulate BPPC redifferentiation

##### 4.3.3.1 FXR

The final step of BEC-mediated liver regeneration is BPPC redifferentiation, which contains two directions: hepatocytes and BECs. Farnesoid X receptor (FXR) is a central factor that controls the redifferentiation of BPPCs to both directions. The regulation of FXR on cell proliferation during liver regeneration has been reported in the CCl_4_- and PH-induced liver injury model (Huang et al., 2006; Meng et al., 2010). In zebrafish, FXR was induced in the BECs upon extreme hepatocyte injury and then reduced to normal levels when regeneration is completed. FXR inhibition would disrupt BPPC redifferentiation, leading to the accumulation of BPPCs and the defects in hepatocyte and BEC regeneration. However, FXR is dispensable for BEC dedifferentiation or BPPC proliferation. FXR controls BPPC redifferentiation to hepatocytes and BECs through transcriptional regulating *erk1* and *notch3*, respectively (Cai et al., 2021). Despite these findings, another group reported that FXR activation may impair BEC-mediated liver regeneration in zebrafish (Jung et al., 2021). However, differing from the prior study that contains both pharmacological and genetic data, the findings in the latter study are mainly based on chemical treatments. Indeed, high doses of FXR agonists caused the apoptosis of BECs and BEC-derived cells, which may be attributed to the toxicological effects beyond FXR. Besides, the lower and appropriate dose of FXR agonist treatments could promote hepatocyte regeneration (Cai et al., 2021), suggesting the requirements of FXR in BEC-mediated liver regeneration.

##### 4.3.3.2 Hdac1

Through chemical screening, the inhibitors of HDAC1/2 and KDM1A were found to impair the redifferentiation of BPPCs into hepatocytes. Because zebrafish only have Hdac1 and Hdac1 protein was highly induced in the BPPCs (Noel et al., 2008; Ko et al., 2019), Hdac1 is thought to be the essential factor of BEC-mediated liver regeneration. Even though the *hdac1* homozygous mutants could not survive to 5 days and the heterozygous mutants exhibited no obvious regenerative phenotype, the authors found that the reduced dosage of HDAC inhibitor would lead to regenerative defects in heterozygous mutants but not in wild-type. During the redifferentiation process, the hyperacetylation of the *sox9b* genomic locus depends on Hdac1 activation, and *sox9b* mutation could rescue the defects of hepatocyte differentiation in *hdac1* heterozygous mutants. On the other hand, Hdac1 controls BPPC-to-BEC redifferentiation by enhancing Notch activity, which is achieved by repressing the Cdk8/Fbxw7-mediated degradation pathway of NICD (Ko et al., 2019). However, whether Hdac1 directly regulates the acetylation of Cdk8/Fbxw7 genomic locus remains unknown.

##### 4.3.3.3 Mdka

The essential roles of VEGF signaling imply the requirements of activated hepatic stellate cells, which could secrete the VEGF and other signaling ligands (Cai et al., 2023). A recently published paper further proved this hypothesis. Midkine (MDK), a heparin-binding growth factor, interacts with a range of cell surface receptors, thereby modulating processes like cell proliferation, migration, and differentiation (Muramatsu, 2010). The expression of *mdka* significantly increased in the stellate cells upon injury, and its receptor *ncl* upregulated in the BECs. Genetic mutation of *mdka* and *ncl* both blocked the redifferentiation of BPPCs into hepatocytes (Zhang et al., 2024). Compared to the transcriptional control of ERK1 by FXR (Cai et al., 2021), Mdka-Ncl signaling regulates BPPC redifferentiation by activating ERK1/2 (Zhang et al., 2024).

##### 4.3.3.4 Bmp

Bone morphogenetic protein (Bmp) signaling seems to be a regulatory factor that balances the redifferentiation to hepatocyte and BEC directions (Choi et al., 2017). Upon extreme hepatocyte loss, those genes involved in Bmp signaling were upregulated at the redifferentiation stages. Bmp inhibition maintained the BPPCs as undifferentiated, thus disrupting the direction of hepatocyte differentiation. However, differing from Hdac1 or FXR inhibition, Bmp inhibition would increase the number of newly regenerative BECs, implying that Bmp signaling may control the hepatocyte differentiation direction to antagonize the BEC direction. The authors claimed that this phenomenon was caused by the abnormal proliferation of BECs. However, we thought that the promotion of BPPC-to-BEC redifferentiation may be one reason to explain this phenotype.

##### 4.3.3.5 Tel2

Tel2 also regulates BPPC redifferentiation in addition to its role in promoting BPPC proliferation. The roles of Tel2 in regulating telomere length and localizing telomeric DNA were first identified in yeast and nematodes (Kota and Runge, 1998; Lim et al., 2001). The *tel2* mutants showed redundant BPPCs and defective hepatocyte regeneration. Interestingly, Tel2 regulates the transcription of *hhex* in BECs and BPPC-to-hepatocyte redifferentiation independent of telomere-related function, and the *hhex* heterozygous mutants showed defective BPPC-to-hepatocyte redifferentiation (Zhang et al., 2022).

##### 4.3.3.6 Wnt and Notch

By combining the Mtz/NTR system with alcohol exposure, BECs are proved to be the central resource of hepatocyte regeneration in zebrafish fibrotic livers (Huang et al., 2014). Consistent with its roles in liver development, Wnt signaling is found to be required for BPPC-to-hepatocyte redifferentiation and antagonizes the Notch signaling to ensure the hepatocyte direction in BEC-mediated liver regeneration (Huang et al., 2014; Russell et al., 2019a). Notch signaling is critical for both BEC development and regeneration (Zong et al., 2009; Ko et al., 2019; Cai et al., 2021). The mutation of *notch3* completely blocks the redifferentiation of BPPCs into BECs (Ko et al., 2019). Moreover, Notch inhibition would promote the BPPC-to-hepatocyte redifferentiation through repressing *sox9b* (Russell et al., 2019a), thus confirming the roles of Notch and Sox9b in BPPC-to-BEC differentiation direction (Ko et al., 2019). Hence, Wnt and Notch signaling may be the key effectors that control hepatocyte direction and BEC direction, respectively.

##### 4.3.3.7 Dnmt1

DNA methylation was continuously maintained in the BECs during BEC-mediated liver regeneration, which may be the reason why Dnmt1 is also involved in BPPC redifferentiation (He et al., 2022). DNA methylation inhibition at later regenerative stages also released the DNA methylation in the *p53* genomic locus and increased the transcription of *p53*, leading to the BPPC accumulation and BPPC redifferentiation defects. Besides, the activation of Bmp signaling would be blocked upon late DNA methylation inhibition. *p53* has been reported to be a regulator of BMP signaling (Liu et al., 2013; Balboni et al., 2015). Thus, the authors thought that *p53* activation contributes to the Bmp repression after DNA methylation inhibition.

##### 4.3.3.8 Other regulatory factors

In addition to these factors, the MRN complex is reported to prevent the BEC-derived hepatocytes from apoptosis through the ATR-Chk1 pathway. Either *rad50* or *nbn* mutation would activate DNA damage response and thus trigger apoptosis in BEC-derived hepatocytes (Song et al., 2023). Signal transducer and activator 3(Stat3) has been reported to be involved in hepatocyte proliferation-mediated liver regeneration (Moh et al., 2007) and BEC-derived oval cell proliferation (Sánchez et al., 2004). Like the roles of Tel2, Stat3 is crucial for the BPPC proliferation and redifferentiation. However, despite the BPPC-to-hepatocyte direction was blocked, the BPPC-to-BEC direction seemed to be unaffected upon Stat3 inhibition (Khaliq et al., 2018). Epithelial cell adhesion molecule (EpCAM) is dispensable for BPPC formation, proliferation, and redifferentiation but regulates the maturation and reconstruction of biliary network (Lee et al., 2024). Those factors found in zebrafish BEC-mediated liver regeneration provide many insights into mammal liver recovery (Figure 3). The roles of Wnt and Notch were validated in mouse models (Pu et al., 2023), and VEGF overexpression was reported to promote the BEC-to-hepatocyte transdifferentiation and thus accelerate liver recovery upon chronic liver injury in mice (Rizvi et al., 2023). Even though these studies reported in recent years have deepened our understanding of BEC-mediated liver regeneration, the regulatory mechanisms remain largely unknown, especially how BECs sense the deficiency of hepatocyte proliferation and then start to trans-differentiate upon acute and chronic liver injury.

## 5 Acetaminophen-induced liver injury in zebrafish

Utilizing hepatotoxic over-the-counter medications such as acetaminophen (APAP), tetracycline, erythromycin, aspirin, amiodarone, and cyclosporine A, it has been established that drug-induced liver injury manifests similarly in both zebrafish and humans (Shimizu et al., 2023). Unlike those drugs frequently used to assess hepatotoxicity in zebrafish, APAP could also be used to study liver regeneration after its acute injury to the liver (Driessen et al., 2015; Yoon et al., 2016).

APAP produced similar effects on embryonic and larval hepatocytes, and APAP diminished liver size in a dose- and time-dependent fashion in larval zebrafish (North et al., 2010). The larval zebrafish was often used to assess liver regeneration upon APAP-induced liver injury. 10 mM APAP was used to treat zebrafish larvae from 3.5 to 5 dpf, and the regenerative effects were checked at 2 days after APAP exposure. Hepatocyte proliferation, but not BEC transdifferentiation, contributes to liver regeneration after APAP-induced liver injury (Russell et al., 2017). Using the APAP-induced liver injury model, the chemicals PGE2 and NAC were found to reduce the toxicological effects of APAP and promote liver regeneration after APAP exposure (North et al., 2010). Similar to BEC-mediated liver regeneration, BET inhibition would reduce hepatocyte proliferation in the zebrafish APAP-induced injury model (Russell et al., 2017), indicating the conserved roles of BET proteins in both hepatocyte- and BEC-mediated liver regeneration. By performing SLAM-ITseq, the nascent transcriptome was investigated during the initiation of liver injury and regeneration after APAP exposure. A swift metabolic shift from the postprandial to the fasting state was observed, leading to the induction of the nuclear erythroid 2-related factor (Nrf2) antioxidant program. Activation of Nrf2 in hepatocytes is crucial for the initiation of the pentose phosphate pathway (PPP), thereby enhancing liver regeneration and survival in cases of APAP-induced liver injury (Tan et al., 2024). Additionally, pharmacological activation of FXR could promote liver regeneration after APAP exposure, confirming the roles of FXR in hepatocyte proliferation-mediated liver regeneration cross species (Huang et al., 2006; Jung et al., 2021). Although APAP could be used to study zebrafish liver regeneration, this model is not commonly used due to the vast researches in mouse models.

## 6 Other liver regeneration models in zebrafish

### 6.1 Chemoptogenetic liver injury model

Optogenetic approaches offer unprecedented precision in spatially and temporally regulating tissue manipulation, enabling minute control over biological processes (Portugues et al., 2013). To create a chemoptogenetic hepatocyte ablation tool for extended live imaging, the transgenic line Tg(fabp10a:dL5**-mCer3), abbreviated *Tg(LiverZap)*, was generated. In this model, the exposure of 12 min NIR light would induce cell death of hepatocytes by producing ROS in larval zebrafish. Eight hours after the injury, apoptotic activity was observed in the liver region. Hepatocyte ablation was categorized into two distinct types: mild (30%) and severe (70%). Mildly ablated livers regenerated within 3 days post-injury, while severely ablated livers recovered within 7 days. Notably, the mechanisms of liver regeneration differed between the two conditions. Mildly ablated livers regenerated primarily through hepatocyte proliferation, whereas severely ablated livers were repaired via transdifferentiation of BECs. Additionally, using the LiverZap tool, the researchers demonstrated that targeted ablation of hepatocytes in a discrete region of interest is unexpectedly effective in triggering BEC-mediated regeneration, thereby challenging current perspectives on liver progenitor cell activation (So et al., 2020). Dynamic rearrangement of the biliary network and E-cadherin re-localization could both be observed in this model, indicating that cell adhesion modulation may be a pivotal step in BEC-mediated liver regeneration (Ambrosio et al., 2024). This model expands the current regeneration toolkit and enables detailed analysis of critical cellular dynamics, which are essential for understanding how the liver’s complex architecture is deconstructed during injury and reconstructed during repair. However, due to the variability in regeneration speed and dependency on injury severity, this model is limited in its ability to investigate the key factors involved in hepatocyte proliferation or BEC-mediated liver regeneration.

### 6.2 Oncogene overexpression-induced liver injury model

To identify small molecules that can facilitate liver progenitor cell (LPC)-to-hepatocyte differentiation, the *Tg(fabp10a:pt-β-catenin)* zebrafish transgenic line was constructed for LPC-mediated liver regeneration in which a mutated, stable form of *Xenopus* β-catenin is overexpressed in hepatocytes (So et al., 2021). The pt-β-catenin variant includes four point mutations (S33A, S37A, T41A, and S45A) at putative phosphorylation sites, and these mutations activate β-catenin by preventing its phosphorylation and subsequent degradation (Evason et al., 2015). Zebrafish hepatocyte-specific overexpression of pt-β-catenin would cause hepatocellular carcinoma (HCC) and recapitulate the pathologic features of human HCC (Evason et al., 2015). As early as 7 dpf, the hepatocytes of *Tg(fabp10a:pt-β-catenin)* larvae exhibited DNA damage, apoptosis, and senescence, which would be partially recovered at 30 dpf. The lineage tracing data showed that liver regeneration was achieved by both hepatocyte and BEC contributions. Thus, this model differs from the Mtz/NTR model. Using this model, the EGFR-ERK-Sox9 axis was found to play opposing roles during LPC-mediated liver regeneration, and the treatment of EGFR and ERK inhibitors would accelerate the differentiation of hepatocytes from LPCs (So et al., 2021). The inhibitory role of Sox9 in hepatocyte differentiation has also been observed in BEC-mediated liver regeneration, suggesting a conserved function across different models. Notably, EGFR and ERK signaling pathways exhibit opposing roles in BEC- and LPC-mediated liver regeneration (Cai et al., 2021; So et al., 2021; Oderberg and Goessling, 2023), highlighting the distinct regenerative mechanisms employed in different liver injury models. Additionally, PPARα activation is found to augment the differentiation of LPCs to hepatocytes by suppressing YAP signaling in this model (Kim et al., 2023).

### 6.3 Hepatic cryoinjury model

A recent paper published in *Development* reports a new liver injury model in which liver cryoinjury is induced by adapting the CUBIC tissue-clearing approach in adult zebrafish. The ventral lobe was selected for cryoinjury due to its surgical accessibility. To ensure a reproducible and consistent injury, all procedures were performed at the level of the anterior fins and towards the midline. By 14 days post-cryoinjury, the injured area was nearly fully repaired. The cryoinjury would induce a localized necrotic and apoptotic lesion characterized by inflammation and infiltration of innate immune cells. Following the initial phase, the liver would suffer from fibrosis, which then be resolved by liver regeneration within 30 days. Cryoinjury would induce both localized and distal compensatory hyperplasia through trigger cell proliferation. The transcriptional landscape following cryoinjury has also been discovered (Sande-Melon et al., 2024). However, despite this, more information about this model needs further investigation, especially the functional validation of the regulatory factors.

## 7 Discussion

### 7.1 Zebrafish as the ideal model to study BEC-mediated liver regeneration

After discovering BEC-mediated liver regeneration in zebrafish, this type of liver regeneration was also found in several mouse liver injury models. Three years after the zebrafish studies were published, by performing hepatocyte-specific knock-out of β1-integrin or overexpression of p21 combined with DDC/CDE/MCD-induced liver injury in mice, 15%–25% of the newly regenerated hepatocytes were found to be derived from BECs. However, the total experimental periods of this model reach about 2 months, and the contribution of BECs to hepatocytes is relatively low (Raven et al., 2017). Using the TAA/DDC-induced chronic liver injury model in mice, another group reported that chronic liver injury would also generate BEC-derived hepatocytes. Upon chronic injury for 6 months, about 10% of new hepatocytes were derived from BECs. These BEC-derived hepatocytes own a high proliferating capacity, and the ratio of these cells would reach 55% upon liver injury for 13 months (Deng et al., 2018). Besides, hepatocyte-specific deletion of β-catenin in mice could also trigger the BEC-to-hepatocyte transdifferentiation upon CDE-induced liver injury for 2 weeks. Statistically, about 20% of hepatocytes come from BEC transdifferentiation after liver recovery for 2 weeks, and this ratio reaches 70% after 6 months of recovery, confirming the proliferative capacity of BEC-derived hepatocytes (Russell et al., 2019b). Long-term CCl_4_ treatment could also trigger BEC-derived hepatocyte regeneration in mice. However, like all the other models above, the contribution ratio of BECs is low, only up to 13% after 4 weeks of recovery upon CCl_4_-induced injury for 16 weeks (Manco et al., 2019). Thus, these mouse models have similar disadvantages, including long experimental periods, operating difficulty, and low contribution ratio.

Compared to the mouse models, almost all newly regenerated hepatocytes are attributed to the BEC transdifferentiation in the zebrafish model (Choi et al., 2014; He et al., 2014). The Mtz/NTR system ensures extreme hepatocyte loss in zebrafish, thus providing a more specific model to study BEC-mediated liver regeneration. Additionally, the zebrafish larvae at 5 dpf could be used to induce liver injury, and the regeneration process can be completed as quickly as 48 h after Mtz treatment (He et al., 2014; Cai et al., 2023). Therefore, the experimental period of the zebrafish model is much shorter than that of the mouse models. This advantage provides the zebrafish BEC-mediated liver regeneration model as ideal for drug screening and regulatory factor exploration.

### 7.2 The difference and similarity between zebrafish and mammal liver regeneration

The process of liver regeneration via hepatocyte proliferation seems to be conserved, as the majority of hepatocytes in the resected lobe of zebrafish begin to proliferate within 2–3 days following partial hepatectomy (Kan et al., 2009). Research in zebrafish has identified multiple mechanisms that control liver regeneration, which is similar to mammals. However, the difference exists between zebrafish and mammals. In mammals, the hepatocytes have been proven to be heterogeneous by several genetic lineage tracing and single-cell sequencing studies (Aizarani et al., 2019). By using lineage tracing from the telomerase reverse transcriptase (Tert) locus in mice, rare hepatocytes with high telomerase expression are found to be distributed throughout the liver lobule. Upon injury, the repopulating activity of these Tert^high^ hepatocytes accelerates. When Tert^high^ hepatocytes are genetically ablated in combination with chemical-induced liver injury, there is a marked increase in stellate cell activation and fibrosis (Lin et al., 2018), suggesting the contribution of these cells in liver regeneration. Additionally, Axin2^+^ pericentral hepatocytes may contribute to liver homeostasis and repair (Wang et al., 2015) despite the controversial conclusion (Sun et al., 2020). These studies adequately prove the heterogeneity of hepatocytes in mammalian livers. However, no evidence has been reported supporting the hepatocyte heterogeneity in zebrafish. Besides, a systematic analysis of zebrafish liver is lacking to compare the difference between zebrafish and mammals.

Compared to hepatocytes, BECs exhibit similar plasticity in zebrafish, mice, and humans. Upon extreme or chronic hepatocyte injury, BECs similarly have the capacity to transdifferentiate into functional hepatocytes (He et al., 2014; Raven et al., 2017; Gribben et al., 2024). The regulatory factors involved in BEC-mediated liver regeneration seem to be conserved cross species. Wnt signaling controls the redifferentiation of BPPCs into hepatocytes in both mice and zebrafish (Huang et al., 2014; Pu et al., 2023), while Notch signaling also governs the redifferentiation of BPPCs into BECs in mice and zebrafish (Ko et al., 2019; Cai et al., 2021; Pu et al., 2023). Similar roles of FXR, Hdac1, VEGF, Mdka, and BET have been proved in mouse and zebrafish BEC-mediated liver regeneration models (Ko et al., 2016; Ko et al., 2019; Cai et al., 2021; Cai et al., 2023; Rizvi et al., 2023; Zhang et al., 2024). Besides, the PI3K-AKT-mTORC1 pathway regulates the BEC-to-hepatocyte plasticity in both zebrafish and humans (He et al., 2019; Cai et al., 2023; Gribben et al., 2024).

Structural and cellular differences in the liver between mammals and zebrafish may lead to distinct regenerative responses. In zebrafish, the bile duct structure is defined by a single layer of cuboidal cells, which is specific to hilar bile ducts and not present in peripheral bile ducts (Cai et al., 2021). The lumen formation of peripheral bile ducts typically occurs through the fusion of cytoplasmic vesicles between adjacent biliary cells (Lorent et al., 2010), a process distinct from that in mammals. This unique structure of the zebrafish liver may account for the rapid response of BECs to hepatocyte injury, potentially explaining the high frequency of BEC-mediated liver regeneration observed in zebrafish injury models (He et al., 2014; Ambrosio et al., 2024). Furthermore, zebrafish lack the liver-resident macrophage Kupffer cell, which is found in mammals (Goessling and Sadler, 2015). In mammals, Kupffer cells secrete pro-inflammatory cytokines upon liver injury, which are crucial for hepatocyte proliferation and liver regeneration (Wang et al., 2024). The absence of Kupffer cells in zebrafish may explain the lack of inflammatory factors involved in BEC-mediated liver regeneration and could influence the preferred pathway for liver regeneration.

### 7.3 Studies in zebrafish liver regeneration provide potential therapeutic targets for human liver diseases

Liver transplantation is the only way to treat end-stage liver diseases, which have high mortality (Fisher, 2017). However, the shortage of organ donors and graft rejection limit the application of liver transplantation to many patients (Fisher, 2017). In patients with end-stage liver diseases, hepatocyte proliferation is compromised due to liver steatosis, inflammation, fibrosis, and cirrhosis (Stanger, 2015). Thus, the facilitation of hepatocyte proliferation-mediated liver regeneration seems to be infeasible for liver repair in these cases. Ductular reaction, reflected by BEC proliferation and progenitor activation, is the hallmark of almost all chronic and acute liver diseases (Sato et al., 2019). After the findings of BEC-mediated liver regeneration, promoting the BEC-to-hepatocyte transdifferentiation was postulated to be the alternative way to alleviate severe hepatocyte injury in end-stage liver diseases.

Using the zebrafish extreme liver injury model, VEGF signaling was found to be essential for BEC-to-hepatocyte transdifferentiation (Cai et al., 2023; Rizvi et al., 2023). Based on this phenomenon, another group explored the clinical benefit of VEGF activation in promoting liver repair and restoring liver function in several mammal liver injury models. Using the mRNA-lipid nanoparticles (LNP) delivery system, the controllable transient VEGFA expression in the liver could be archived (Maestro et al., 2021). VEGF mRNA-LNP robustly induced the BEC-to-hepatocyte transdifferentiation and promoted the recovery of liver function in both chronic and acute mouse liver injury models (Rizvi et al., 2023), suggesting the potential clinical benefits of VEGFA mRNA-LNP to alleviate liver diseases. In the livers of patients with end-stage liver diseases, intermediate hepatocyte-like cells exist and express both hepatocyte and BEC markers. Moreover, the presence of these cells is associated with the expression of VEGF receptor KDR (Rizvi et al., 2023), implying that VEGF activation may also stimulate BEC-mediated liver regeneration for human liver disease intervention. Additionally, even though there is a lack of data from mammals, the supplement of either 17 β-estradiol (Chaturantabut et al., 2019), FGF21 protein (Qiang et al., 2021), or PPARα agonist (Kim et al., 2023) could promote the transdifferentiation of BECs to hepatocytes in zebrafish. These findings suggest that using zebrafish as the model of BEC-mediated liver regeneration could indeed provide a potential translational impact on human health.

### 7.4 Zebrafish models combined with *in vitro* organoids for studying liver regeneration

Although significant progress has been made in studying liver regeneration using zebrafish models, a comprehensive understanding of hepatic biology and disease pathology requires the integration of diverse research tools. The development of liver organoids, a self-organizing and self-renewing three-dimensional cell culture model, has significantly advanced liver research. Liver organoids offer a physiologically relevant platform for drug screening and development, personalized medicine, disease modeling, and the study of liver regeneration (Chawla and Das, 2023). Recently, liver organoids have been used to alleviate liver damage and promote liver repair in mice (Aloia et al., 2019; Yuan et al., 2024), indicating the huge therapeutic potential for liver diseases. However, the emergence of organoid technology is accompanied by several limitations that must be addressed, including the high cost of these models, limited availability of source tissues, and the need for multilineage liver organoids to accurately replicate the cellular heterogeneity of the liver (Chawla and Das, 2023). In contrast, zebrafish offer several advantages, including low cost, high reproductive capacity, and the ability to replicate an *in vivo* microenvironment. Therefore, considering the distinct benefits of zebrafish models and organoids, integrating these tools is recommended for studying liver regeneration, particularly in pre-clinical research.

## 8 Conclusion and perspective

In conclusion, the utilization of the zebrafish as an animal model in liver regeneration studies has significantly contributed to the progression and advancement of this research field. The advantages of zebrafish make it suitable for small molecule and drug screening, providing new insights for treating human liver diseases. The scope of hepatobiliary diseases being investigated in zebrafish models is rapidly expanding. Alongside this, novel tools and methodologies are being formulated, encompassing precision gene editing techniques to introduce mutations in specific genes, sophisticated imaging techniques to monitor hepatic cells, and new sequencing methods (such as scRNA-seq and stRNA-seq) to assess the intracellular transcriptional changes upon injury. Studies of liver regeneration using zebrafish models not only enhance our comprehension of the underlying mechanisms of liver diseases but also aid in identifying novel therapeutic targets and potential candidate compounds for treating liver diseases.
